# Genetic transformation of sweet oranges to over-express *SABP2 *gene

**DOI:** 10.1186/1753-6561-8-S4-P109

**Published:** 2014-10-01

**Authors:** Lísia Borges Attílio, Polyana Kelly Martins, Laura Melissa Gómez-Krapp, Marcos Antônio Machado, Juliana Freitas-Astúa

**Affiliations:** 1 Centro de Citricultura Sylvio Moreira, Instituto Agronômico de Campinas, Cordeirópolis, SP, Brazil; 2 EMBRAPA Agroenergy - Estação Parque Biológico, Brasilia, DF, Brazil; 3 EMBRAPA Cassava & Fruits, Cruz das Almas, Bahia, Brazil Centro de Citricultura Sylvio Moreira, Instituto Agronômico de Campinas, Cordeirópolis, SP, Brazil

## Background

The history of the world citrus industry is marked by a series of diseases caused by different etiologic agents. Several of them are characterized as biotrophic pathogens like *Xanthomonas citri *subsp. *citri*, three *Candidatus *Liberibacter spp and *Citrus leprosis virus C *(CiLV-C). In plant-pathogen interactions, the role of salicylic acid (SA) in activating defense related genes is well recognized [1]. The SABP2 (Salicylic acid-binding protein 2) is required to convert methyl salicylate to SA as part of the signal transduction pathway that activates systemic acquired resistance, induces PR expression, and enhances disease resistance. Due to the reduced or absence of genetic resistance to these pathogens in commercial sweet orange cultivars, the genetic transformation to over-express a gene involved in the defense response of plants is a possible alternative to produce tolerant or resistant plants to biotrophic pathogens.

## Methods

The aim of this study was to produce 'Hamlin' sweet orange (*Citrus sinensis *L. Osb.) transgenic plants, via *Agrobacterium tumefaciens*, over-expressing the *SABP2 *gene from sweet orange driven by the constitutive promoter ubiquitin (*Ubq10*). The *SABP2 *gene was cloned into pCambia 2301 and inserted into *A. tumefaciens *EHA 105. The genetic transformation was performed using epicotyl segments from seedlings [2].

## Results and conclusions

A total of 620 explants in three independent experiments were introduced and approximately 336 shoots were regenerated. The GUS histochemical test was performed and confirmed the transformation of 30 positive shoots. These shoots are being grafted onto Carrizo citrange [*Citrus sinensis *x *Poncirus trifoliata *(L.) Raf.] seedlings grown in test tubes containing MS culture medium. The presence of the transgene will be evaluated by PCR using specifics primers that amplify part of the ubiquitin promoter and part of the gene. *SABP2 *expression levels in transgenic plants will be assessed through qPCR. After bud multiplication, transgenic plants will be evaluated for their response to citrus canker, HLB and leprosis.
